# Traditional Wild Food Plants Gathered by Ethnic Groups Living in Semi-Arid Region of Punjab, Pakistan

**DOI:** 10.3390/biology12020269

**Published:** 2023-02-08

**Authors:** Muhammad Waheed, Shiekh Marifatul Haq, Fahim Arshad, Rainer W. Bussmann, Andrea Pieroni, Eman A. Mahmoud, Ryan Casini, Kowiyou Yessoufou, Hosam O. Elansary

**Affiliations:** 1 Department of Botany, University of Okara, Okara 56300, Pakistan; 2 Department of Ethnobotany, Institute of Botany, Ilia State University, 0162 Tbilisi, Georgia; 3 Department of Botany, State Museum of Natural History, Erbprinzenstrasse 14, 76133 Karlsruhe, Germany; 4 University of Gastronomic Sciences, Piazza Vittorio Emanuele II, 9I-12042 Pollenzo, Italy; 5 Department of Medical Analysis, Tishk International University, Erbil 44001, Kurdistan, Iraq; 6 Department of Food Industries, Faculty of Agriculture, Damietta University, Damietta 34511, Egypt; 7 School of Public Health, University of California, Berkeley, 2121 Berkeley Way, Berkeley, CA 94704, USA; 8 Department of Geography, Environmental Management, and Energy Studies, University of Johannes-Burg, APK Campus, Johannesburg 2006, South Africa; 9 Department of Plant Production, College of Food & Agriculture Sciences, King Saud University, P.O. Box 2460, Riyadh 11451, Saudi Arabia

**Keywords:** wild foods plants, local cultures, indicator analysis, Indo–Pak border

## Abstract

**Simple Summary:**

In traditional food systems, especially for rural populations around the world, wild food plants have remained crucial. These resources must be quickly documented in order to lay the groundwork for sustainable livelihoods and food security. In this study, we looked at 71 traditional food plants and the cultural significances and economic views on the diversity of food plants in the semi-arid region of Punjab, Pakistan, and assessed how local people use wild foods according to preference, seasonality, and availability. We gathered information about 71 key traditional food plants from five ethnic groups (Arain, Jutt, Rajpot, Mewati, and Dogar) by conducting semi-structured interviews, and holding group discussions. In this study, we documented four species (*Capparis decidua*, *Cannabis sativa*, *Salvadora oleoides*, and *Salvadora persica*) which overlapped between all ethnic groups. Seven species did, however, overlap over three indigenous ethnic groups, the Arain, Jutt, and Dogar. More resemblances were seen between the Jutt and Arain ethnic groups due to their extensive historical cohabitation in the same region. *Amaranthus viridis*, *Physalis minima*, *Ziziphus nammularia*, *Chenopodium album*, *Cucumus melo*, and *Ficus palmata* are the food species that we have identified as food indicators and are important in regional diets. This study represents the first effort to compare the use of food plants across cultural groups in the semi-arid area.

**Abstract:**

Wild edible food plants (WFPs) are valuable resources in the traditional food systems of many local cultures worldwide, particularly in underdeveloped regions. Understanding patterns of food preferences requires conducting cross-cultural food studies among various ethnic groups in a specific area. In this context, the current study aimed to record WFP use among five ethnic groups in Punjab, Pakistan, by interviewing 175 informants selected through snowball sampling. The indicator food species for different ethnic groups were calculated using indicator analysis based on the percentage of citations. A total of 71 wild food plants (WFPs) belonging to 57 genera and 27 families were observed in the study area. A high proportion of these wild food plants (WFPs) belonged to Fabaceae with eleven species (15%), followed by Moraceae with seven species (9%). Fruits were most widely used (43%), followed by leaves (19%), and shoots (16%). The majority (35 species, 49%) of plants of WFPs were eaten as cooked vegetables. A cross-cultural comparison revealed that four species overlapped among five ethnic groups (Arain, Jutt, Rajpot, Mewati, and Dogar). The Arain ethnic group gathered and consumed a remarkable number of wild plants (35 species), possibly due to a special connection with the general abundance of the local flora, and being close to nature by adopting professions more allied to WFPs in the study area. The analysis of indicator species revealed distinct significant indicator values (*p* ≤ 0.05) between the main food species among the various ethnic groups. *Amaranthus viridis* was a common indicator of food in all five ethnic groups, while *Ziziphus nammularia* was a common indicator food plant of the Mewati, Rajpot, and Jutt ethnic groups; these plants are important in local diets, especially during times of food scarcity brought on by disease or drought. In addition, the current study reports 20 WFPs that have been rarely documented as human food in Pakistan’s ethnobotanical literature. Future development plans should consider biocultural heritage and pay appropriate attention to local ecological knowledge, dynamics, and historical exchanges of traditional food systems.

## 1. Introduction

Wild food plants have continued to play a significant role in traditional food systems, particularly in rural populations all over the world [1]. However, reliance on wild food plants has significantly declined in many localities as a result of the major sociocultural changes that local populations are experiencing and global climate change [2]. Throughout human history, diets and traditional food systems have included wild food plants because they contain vital nutrients and bioactive substances [3]. Traditional diets from the past and present have proven to provide important health advantages [4].

Wild plants provide food, medicine, and ritual importance for tribal communities. They might also contain more nutrients than their domesticated counterparts [5,6]. Wild food plant parts such as fruits, flowers, roots, and tubers are abundant sources of fiber, proteins, fats, and carbohydrates that can supply the nutrients required for a balanced diet every day [7,8]. Fruit and leaf components are used more frequently, maybe due to the comparatively wide variety of organic and inorganic biomolecule combinations. The adoption of a modern lifestyle has led to a rise in the consumption of ready-to-eat foods (junk food), which raises the risk of illnesses connected to incorrect and unbalanced nutrition [9,10]. The bio-cultural heritage associated with wild food plants and neglected species has been the focus of numerous investigations over the past decade all over the world aimed toward the domestication of new crops [11,12,13].

Food from the Punjab area of Pakistan and India is known as Punjabi cuisine. It has a number of similarities to the cuisine of Kashmir and other nearby states. Punjabi food is varied and depends on the location. A lifestyle based on agriculture and farming that has long been a part of Punjab has affected the regional cuisine, which is supported by locally produced staple foods. While many Punjabi dishes are popular throughout Pakistan, others, such as Sarson saag, shami kebab, tandoori chicken, and Makki di roti, are exclusive to Punjab. Rice, wheat, and dairy products are staple foods. Paneer, butter, clarified butter, and sunflower oil is all utilized. Punjabi cuisine is renowned for its bold and spicy flavors, both in Pakistan and abroad. Its meals, rich in ghee, are considered a challenge for those with a strong palate. A refreshing drink known as lassi, often served as a welcome drink, is commonly enjoyed.

Pakistan, a developing nation, is the eleventh highest-risk country in the world for food insecurity [14]. Nearly 40% of households in northwest Pakistan are considered to be food insecure as a result of the region’s large population and frequent natural and man-made disasters that endanger local means of subsistence and food access [15]. Using wild edible plants as food is a crucial local survival tactic during periods of famine or drought. Threats to the preservation of biodiversity may arise from the unsustainable use of some rare species or plant parts [16]. However, traditional knowledge of wild edible plants is still only partially considered in ethnobotany. Many studies have been conducted on the ethnobotanical and ethnomedicinal aspects of wild edible plants in Punjab [3,7,8,13,14,15,16,17,18,19,20,21,22,23,24,25,26,27,28] but no investigation on wild edible plants (WFPs) has been conducted in the border belt area of the semi-arid region of Punjab, Pakistan. The semi-arid region of Punjab has a variety of wild flora, and the local people are also utilizing them, but the traditional knowledge related to their utilization has not been explored; in this context, the present research is the first study with special emphasis on wild food plants (WFPs) gathered by various ethnic groups of the remote area of the border belt region of Punjab, Pakistan. Therefore, the main research objectives of the study are:

- To investigate and record the variety of wild food plants gathered in the remote areas of the semi-arid region.
- To research and document regional cuisine, culinary practices, and any potential food taboos.
- To assess the gathering and consumption of wild food plants across a variety of ethnic groups in order to comprehend the causes of any similarities and/or differences as well as the dynamics of transportation.

## 2. Materials and Methods

### 2.1. Study Area

**Figure 1:**
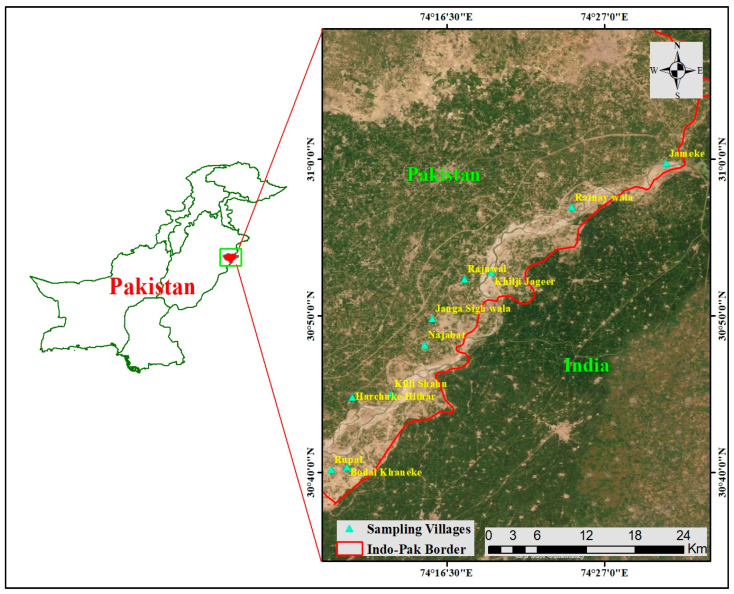
A map showing the location of the study communities from Pakistan’s semi-arid region.

The current study was conducted in Kasur District, a semi-arid region of Punjab, Pakistan. The district Kasur has a total area of 3995 km^2^, and the Ravi and Satluj rivers border it on the north and south, respectively. Its western border is toward the Okara District, its northern border is formed by the Nankana Sahib District, and its eastern and southern borders form the frontier with India. Its northern boundary is formed by the Lahore District. Geographically, the Kasur district is a basin area of the Sutlej riverine and semi-arid plain [29]. The district is separated into two sections: upland (far from the rivers) and low-lying or riverine (near the two adjacent rivers). During the monsoon season, the riverine region frequently floods. This area has a higher water level than the uplands. The upland is made up of level plains with a north–south slope. The location is frequently between 150 and 200 m above sea level (Figure 1). Kasur has a generally mild climate, but summers are hot, with temperatures exceeding 40 °C [30]. The highest temperature is recorded in June, and the lowest temperature in January. Floristically, it belongs to the Sahara Sindian region and is home to a diverse range of plant species. In its open and arid areas, the vegetation includes xerophytic and thermophilic species, but riverine belts support a variety of macrophytes. The land around it is a flat alluvial plain, bounded to the northwest by the Ravi River and to the southeast by the Sutlej River [31]. There are five irrigation canals that run through the district. The main industry is agriculture, and the area grows sugarcane, cotton, wheat, rice, vegetables, and fruits. According to the 2017 census, the total population of District Kasur is 3,454,996, with 1,790,253 men, 1,664,606 women, and 137 transgender people. Rural areas are home to the majority of the people (56.22%). Muslims make up 97% of the overall population, Christians 2%, and Ahmadis, Hindus, and other religions the rest. Kasur’s major castes and tribes are Arain, Jutt, Rajput/Mayo, Dogar, Ansari, Sheikh, and Pathan. The majority of the refugees from East Punjab who settled after the partition with India in this district belong to these tribes and castes. There are also Christians, blacksmiths (Lohar), carpenters (Tarkhan), potters (Kumhar), and barbers (Nai) in the villages. The Arain and Rajput are the most prevalent and strong tribes.

### 2.2. Demographic Information of the Informants

A total of 175 informants belonging to different ethnicities and occupations participated in the study (Table 1). The participants belonged to five different ethnic groups, and of these three were native (Jutt, Dogar, Arain) while two migrated from India to Pakistan in 1947. Most of the respondents were aged between 40 and 60 years (44.57%). Respondents above the age of 60 were few (21.71%) because most older people were ill at the time, and a few had weak eyesight and were not able to identify the specimen. Male participants were 61% and women 38.85%. A majority of informants (42%) were illiterate.

### 2.3. Data Collection

Different field visits, transect walks, and meetings with local people were made from December 2020 to August 2021 to collect data. Through snowball sampling, inhabitants between the ages of 20 and 80 were targeted for the study, with a focus on farmers, herders, and housewives in particular [13]. A survey was conducted with 175 participants, comprising both men and women, from five diverse ethnic and linguistic groups. The interviews were conducted in Urdu (the national language of Pakistan) and Punjabi (the regional language of Punjab). The purpose of the study was explained to the respondents, and we obtained each participant’s verbal prior-informed consent to record and publish their knowledge prior to each interview. Throughout the survey, the Code of Ethics of the International Society of Ethnobiology was strictly followed. Participants were questioned regarding the regional names, components used, collection times, methods of preparation, applications in other dishes, and sales or marketing of the WFP species they have collected in the past and continue to collect today. Additionally, participant observation and open-ended questions were used to collect qualitative ethnographic data. Then, specimens of the cited wild food taxa were gathered from the research area.

Wild food plants specimens were collected, dried, and identified with the help of Flora of Pakistan [32,33,34,35]. The specimens were given botanical and family names and arranged alphabetically. The plant list (http://www.theplantlist.org, accessed on 1 March 2021) and World flora online (http://www.worldfloraonline.org, accessed on 1 March 2021) were used to check the given nomenclature of each taxon. The voucher specimen was deposited in the laboratory of the Department of Botany, the University of Okara, for future reference.

### 2.4. Data Analysis

The WFP data on the species for food purposes gathered from all five castes and ethnic groups were compiled in binary data spreadsheets. To compare ethnobotanical taxa and their applications among five ethnic groups, a Venn diagram was generated using free software (http://bioinformatics.psb.ugent.be/webtools/Venn/, viewed on 18 August 2022). The Chord diagram was created by using origin pro software (version 2021) [12]. The indicator food species among various ethnic groups were computed on the basis of percentage of citation by using PAST software (version 10.3). Ethnobotanists compute JI (Jaccard Similarity Index) to compare renowned data with previously published data from neighboring areas [36]. The following formula is used to calculate JI:

𝐽𝐼 = 𝑐 × 100/(𝑎 + 𝑏) − 𝑐

where “*a*” indicates all species in area A (our study region), “*b*” all species from other published areas, and “*c*” all species that are common to both areas A and B.

**Table 1:** Demography and sociocultural detail of informants.

Ethnic Group		Dogar	Jutt	Arain	Mewati	Rajput
History		An agricultural tribe of Central Punjab	Descendants of Indo-Aryan tribal group native to the Punjab region	The Arain are the descendants of those Arab tribes who came to the region during 8th Century	A racial or ethnic group that originates from the Mewat Muslim Rajput region of western India	Muslim Rajputs are the descendants of Rajputs from the Indian subcontinent’s northern areas.
Language		Punjabi, Urdu	Punjabi	Punjabi, Urdu	Mewati, Urdu	Marwari, Urdu
Subsistence economy		Agriculture, Livestock business, and Services	Agriculture, Pastoralism	Business, Agriculture, and Horticulturalism	Forestry, Agriculture, and Pastoralism	Horticulturalism, Services, Business, and Agriculture
Gender	Male	20	22	20	18	27
	Female	15	18	15	15	5
Education Level	Illiterate	15	20	10	20	9
	Primary	10	12	8	10	11
	Matric	5	6	10	1	6
	Intermediate	2	1	3	4	2
	Graduate	0	2	3	3	2
Age Groups	Between 20 and 40 years	10	11	15	14	9
	Between 40 and 60 years	15	18	20	17	8
	Above 60	12	6	10	4	6

## 3. Results

### 3.1. Diversity of Wild Food Plants and Uses

Overall, 71 wild food plants (WFPs) species belonging to 57 genera and 27 families were documented from remote areas of the Indo–Pak Border in the semi-arid region of Punjab, Pakistan (Table 2). A high proportion of these wild food plants (WFPs) belonged to Fabaceae with eleven species (15.49%), followed by Moraceae with seven species (9.85%), and Cucurbitaceae with five species (7.04%). The abundance and utilization of plant species clearly varied from one ethnic group to others. Asteraceae, Boraginaceae, Malvaceae, and Solanaceae have four species (5.63%) in each. The remaining families had only one or two species each (Table 2).

**Figure 2:**
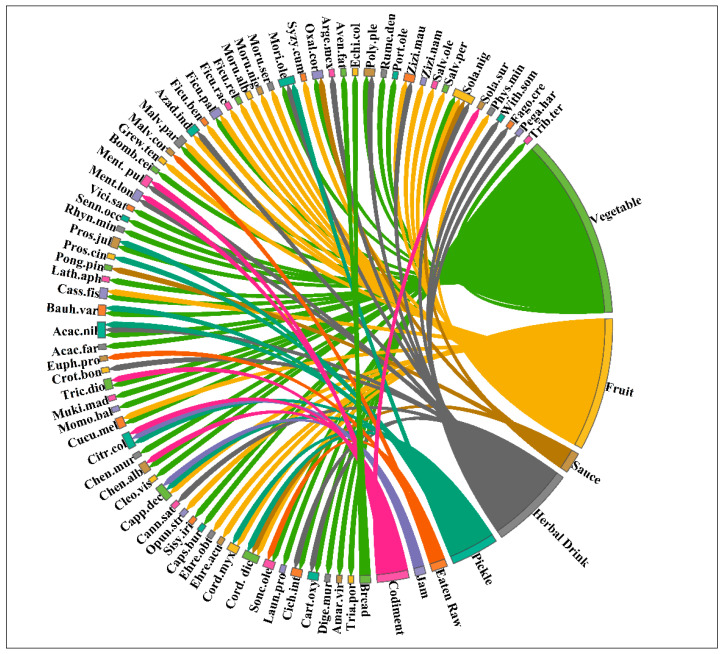
Chord diagram representing the consumption mode and species of recorded WFPs from the Indo–Pak border in Pakistan.

Based on the method of consumption, most WFPs were eaten in the form of cooked vegetables (35 species, 49.29%) (Figure 2). Some important species of this section were *Amaranthus viridis*, *Digera muricata*, *Sisymbrium irio*, *Chenopodium album*, *Trichosanthes dioica*, *Bauhinia variegata*, and *Solanum nigrum*. Similarly, a total of 25 species (35.21%) were eaten as raw ripe fruit; sixteen (22.53%) were used for the preparation of traditional herbal drinks, nine (12.67%) for the preparation of Achar (Pickle); and six (8.45%) as a condiment for flavoring cooked food: (four species for preparation of Chutneys (Sauce), two species for making Maraba (Jam), and two species for traditional bread making) (Figure 2).

**Figure 3:**
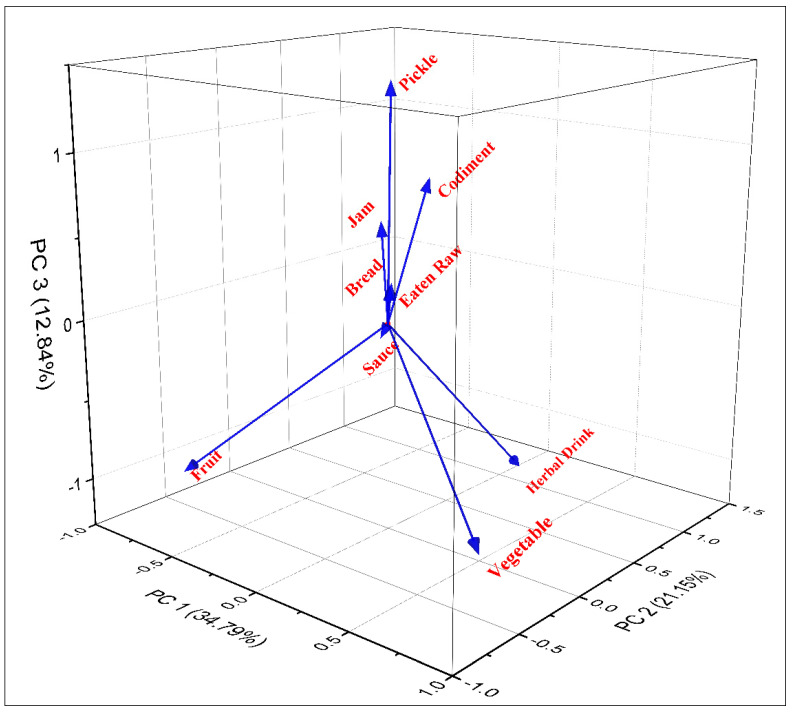
Principal Component Analysis (PCA) biplot of different food categories in the semi-arid region of Indo–Pak border.

The triplot, PC1, PC2, and PC3 explained 68.78% of the mode of consumption of WFPs. Nine clusters of plant consumption modes based on species presence/absence can be seen there: fruit, vegetables, herbal drinks, pickle, condiment, eaten raw, jam, bread, and sauce (Figure 3). PC1 and PC2 show a variation of 13.64 while PC1 and PC3 show a variation of 21.94 and PC2 and PC3 show a variation of 8.31, respectively.

### 3.2. Part Used

**Figure 4:**
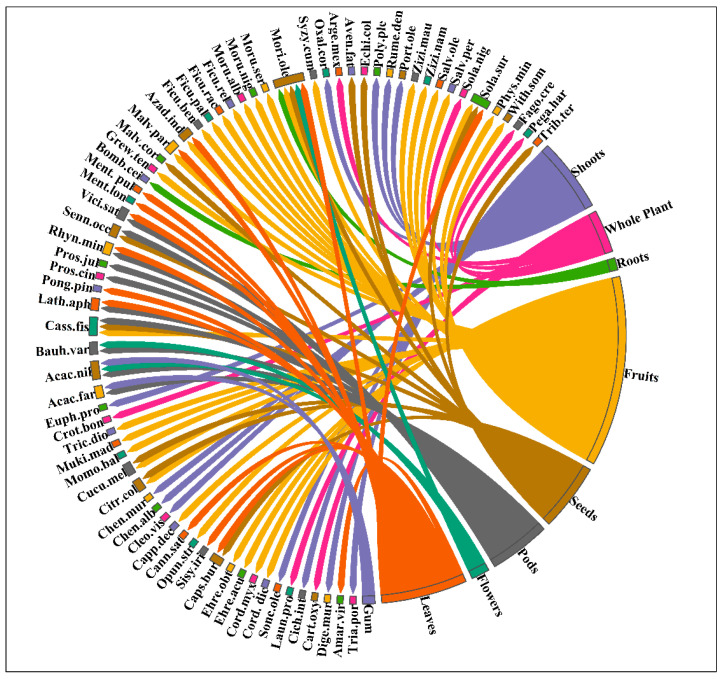
Chord diagram depicting the plant parts used and species name as WFPs.

Fruits were the most widely used plant part (43.66%), followed by leaves (19.71%), shoots (16.90%), seeds (15.49%), pods (14%), whole plant (9.85%), flowers (4.22%), and roots and gum (2.81%) (Figure 4). A majority of WFPs were collected during the monsoon and spring season. According to the study participants, the classification of collected WFPs into their microhabitats (i.e., Scrublands, Forests, Riparian zones, Graveyards, Roadsides, Canals banks, Wetlands, and Sandy places) demonstrates that the distribution sites of wild food plants are well known to the local inhabitants.

**Figure 5:**
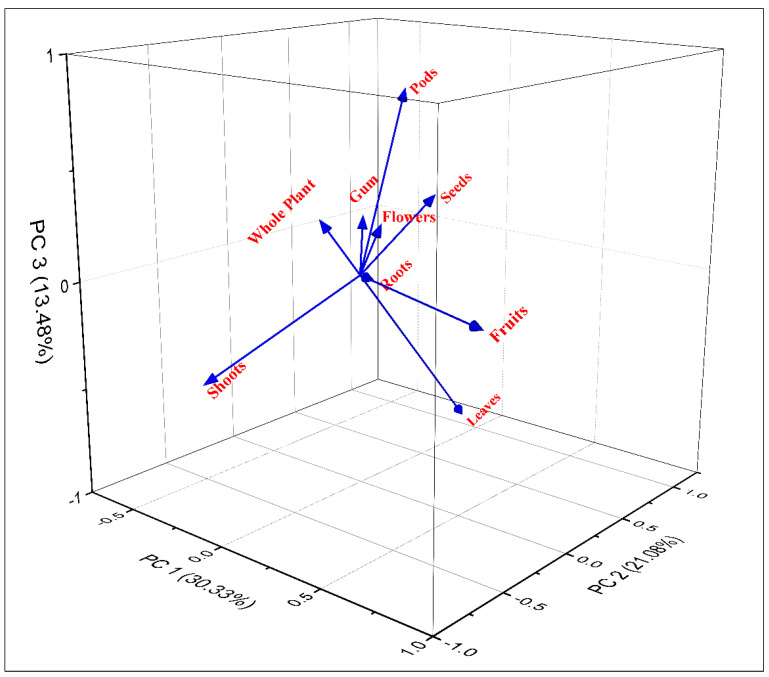
Principal Component Analysis (PCA) biplot of different plant parts used as food by local inhabitants of semi-arid region near Indo–Pak border Pakistan.

In the triplot, PC1, PC2, and PC3 explained 64.89% of the mode of consumption of WFPs. Eight clusters of the plant parts used based on species presence/absence can be seen there: shoots, leaves, seeds, fruit, pods, eaten flowers, whole plant, roots and gum (Figure 5). PC1 and PC2 show a variation of 09.25 while PC1 and PC3 show a variation of 16.85, and PC2 and PC3 show a variation of 7.6, respectively.

### 3.3. WFPs Used as Vegetable

In remote areas of the semi-arid region of Punjab, wild plant species were consumed during periods of food scarcity, or the lean period. Young plant parts, such as roots, stems, and leaves, were prepared using traditional and ethnic methods, either as single species or in combinations. The leaves of the most popular species cooked as vegetables by local participants included *Amaranthus viridis*, *Digera muricata*, *Cichorium intybus*, *Sisymbrium irio*, *Cleome viscosa*, *Chenopodium album*, *Chenopodium murale*, *Mukia madraspatana*, *Oxalis corniculata*, and *Rumex dentatus*. The species that were dried and stored for the future during the drought season included *Amaranthus viridis*, *Chenopodium album*, and *Chenopodium album. Amaranthus viridis*, as it is known locally Chulai, was the most commonly used plant in the district and was thought to be digestive/carminative. Their long growing season, wide availability, and distinct flavor made them one of the most popular vegetable plants. From March through November, local populations gathered the plant from agriculture fields, scrubland, dry places, riparian zones, and lakes. It was popular due to its distinct flavor and accessibility.

### 3.4. WFPs Used as Fruit

It was observed that especially local communities with a herding lifestyle consumed sweet fruits as raw snacks. In total, 25 WFPs were collected from the wild and eaten raw. The most important species were *Cordia dichotoma*, *Cordia myxa*, *Ehretia acuminata*, *Opuntia stricta*, *Ficus palmate*, *Morus alba*, *Morus nigra*, *Ziziphus mauritiana*, *Ziziphus nammularia*, *Salvadora oleoides*, *Salvadora persica*, and *Physalis minima. Ziziphus* and *Morus* were both popular species among all ethnic groups and were collected during March and April.

### 3.5. Herbal Drinks

In our study area, 16 taxa were used to make drinks such as herbal teas, coffees, decoction, and cold drinks. The shoots of *Carthamus oxyacantha* and leaves of *Cichorium intybus* are prepared as herbal teas. Fresh leaves of *Cannabis sativa* are collected and grinded with opium poppy seeds and almonds for preparing Bhang (a traditional cold drink). The gum of *Acacia nilotica* is mixed with water and sugar for preparing herbal drinks while the flowers are boiled in water for preparing herbal tea. The leaves of the *Mentha* species are used for preparing cold drinks and herbal decoctions as medicine. A herbal tea/decoction called “joshanda” was prepared from the dried fruit of the *Ziziphus* species.

### 3.6. Pickles and Condiments

Nine species were processed and preserved in the form of pickles included *Cordia dichotoma, Cordia myxa, Capparis decidua, Citrullus colocynthis, Acacia nilotica, Bauhinia variegata, Prosopis cineria, Prosopis juliflora,* and *Moringa oleifera*. Participants were asked if they gathered wild food plants (WFPs) for commercial purposes during the interviews. The informants reported three species (*Cordia myxa, Citrullus colocynthis*, and *Moringa oleifera*) that were available from the local market as pickles. Out of 71, six species (*Chenopodium album, Citrullus colocynthis, Trichosanthes dioica Mentha longifolia, Mentha pulegium,* and *Solanum surattense*) were used as condiments for improving the taste of the food.

### 3.7. Traditional Cuisine Recipes of WFPs

Several recipes for preparing WFPs were documented. Local people employed WFPs in a number of ways, and an understanding of these species and their eating approaches was passed over generations. Various methods for preparing wild foods were used, depending on the type of the plant. Small pieces of the wild vegetables were sliced and then boiled in water. To prepare cooked food, the raw material was boiled and then fried in oil or butter with onion, garlic, ginger, and green chile. The wild vegetables boiled in water and fried in oil included *Trianthema portulacastrum*, *Amaranthus viridis*, *Digera muricata*, *Cichorium intybus*, *Launaea procumbens*, *Sonchus oleraceus*, *Capsella bursa-pastoris*, *Sisymbrium irio*, *Chenopodium murale*, *Momordica balsmina*, *Trichosanthes dioica*, *Acacia farnesiana*, *Bauhinia variegate*, and *Portulaca oleracea.* The most popular traditional cuisine was Saag prepared by cooking the green leaves of vegetables of the area. The essential ingredient in the recipe of Saag was *Brassica campestris* and *Spinacia oleracea* which with other wild vegetables were boiled in water. After being cooked, their paste was fried in oil or butter with onion, garlic, ginger, and green chili with spices to make the traditional dish (a traditional method of frying). Saag was served with roti (bread) made from corn flour and Lassi (yogurt drink) on special occasions. One species (*Peganum harmala*) was used to make sweet dishes by boiling it in milk with sugar, and two species (*Capparis decidua* and *Citrullus colocynthis*) were used to make Marba (Jam). Traditional bread was made from the flour of two species (*Avena fatua* and *Echinochloa colona*).

### 3.8. Cross Ethnic Comparison

**Figure 6:**
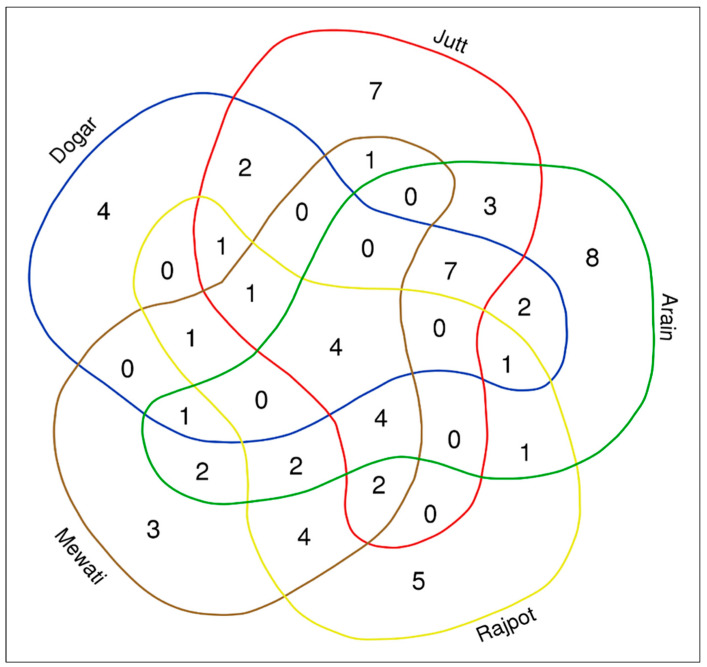
Venn diagram depicting the overlap of quoted wild edible plants among the five studied ethnic groups.

Communities who live in or near the border belt of the semi-arid region still practice ancient cultural customs involving the use of wild plant species that are edible. In this study, we examined how people use wild foods according to preference, seasonality, and cultural availability. Four species (*Capparis decidua*, *Cannabis sativa*, *Salvadora oleoides*, and *Salvadora persica*) were overlapping between all ethnic groups. Seven species did, however, overlap in three indigenous ethnic groups, the Arain, Jutt, and Dogar. The Arain ethnic group gathered and consumed a wide range of wild plants (35 species), most likely as a result of increased population density in the research area, increased knowledge due to the social contacts that resulted, and adoption of occupations more closely related to WFPs. A total of eight species including *Digera muricata*, *Launaea procumbens*, *Momordica balsmina*, *Cordia dichotoma*, *Ficus palmate*, *Solanum surattense*, *Azadirachta indica*, and *Trichosanthes dioica* were idiosyncratic by the Arain ethnic group. As a result, Arain was the leading ethnic group that had most traditional knowledge related to WFPs. Seven idiosyncratic WFPs (i.e., *Carthamus oxyacantha*, *Sonchus oleraceus*, *Citrullus colocynthis*, *Trianthema portulacastrum*, *Argemone Mexicana*, *Polygonum plebeium*, and *Sisymbrium irio*) were only used by Jutt, while the Rajpot ethnic group used five idiosyncratic WFPs including *Mentha pulegium*, *Bauhinia variegate*, *Acacia farnesiana*, *Bombax ceiba*, and *Oxalis corniculata*. The Dogar ethnic group collected four idiosyncratic species (i.e., *Cleome viscosa*, *Malvastrum coromandilianum*, *Physalis minima*, and *Rhynchosia minima*) as food sources (Figure 6). Similarly, the Mewati ethnic group had only three idiosyncratic species including *Ehretia acuminata*, *Ehretia obtusifolia*, and *Capsella bursa-pastoris*. The wide range of sociocultural variances among the studied ethnic communities, which are dispersed across geographic regions in the study area, could be linked to variations in the reported species’ uses.

**Figure 7:**
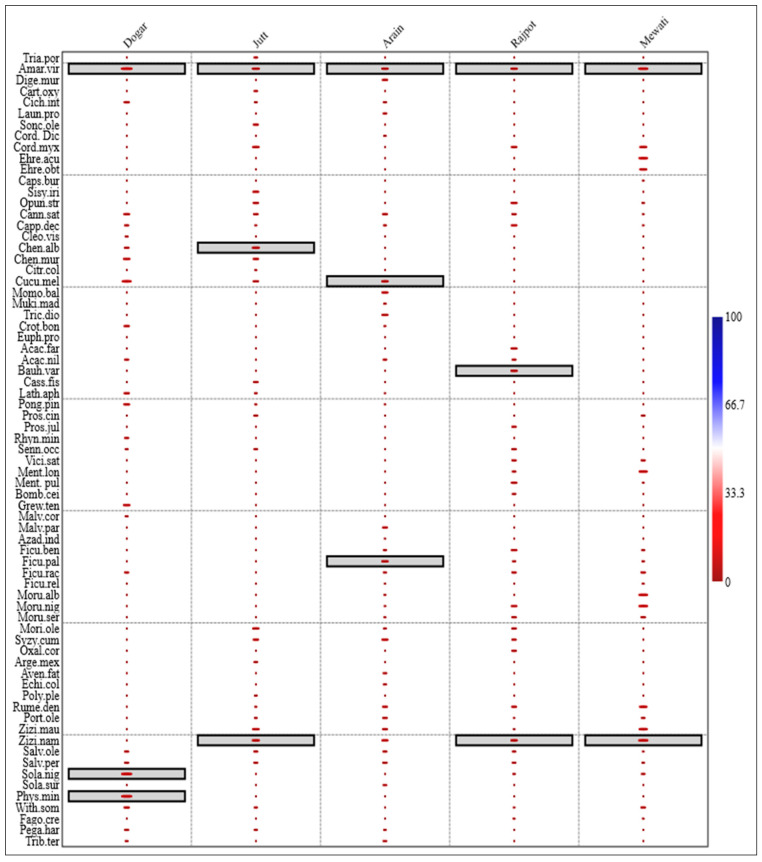
Indicator food species in different ethnic groups in the semi-arid region of Punjab Pakistan. Indicator food plants are highlighted in boxes in different groups. *Amaranthus viridus* was a common indicator species for all ethnic groups, while *Ziziphus nummularia* was a common indicator species for three ethnic groups (Jutt, Rajput, and Mewati). The Y-axis represents the species code (for full species name see Table 2) and the colors represent the indicator value of species in different ethnic groups.

This was further supported by the indicator species analysis that revealed a clear distinction between key food species in different ethnic groups. In the Dogar ethnic group, *Amaranthus viridis*, *Solanum nigrum*, and *Physalis minima* had significant indicator values (*p* ≤ 0.05), while in the Jutt ethnic group *Amaranthus viridis*, *Ziziphus nammularia*, and *Chenopodium album* had significant *p*-values. *Cucumus melo*, *Amaranthus viridus*, and *Ficus palmata* were indicator species of the Arain ethnic group. In the Rajpot ethnic group, indicator food species were *Bauhinia variegata*, *Ziziphus nammularia*, and *Amaranthus viridis* while in the Mewati ethnic group, *Amaranthus viridis* and *Ziziphus nammularia* were indicator food species. *Amaranthus viridis* was a common indicator of food between all five ethnic groups while *Ziziphus nammularia* was a common indicator food plant of the Mewati, Rajput, and Jutt ethnic groups (Figure 7).

### 3.9. Novelty Index of Current Documentation

A comparison between the current findings and published ethnobotanical literature was performed. The documented 71 wild fruits and vegetables of this study were cross verified with 19 published articles of Pakistan (Table 3). This indicated that profound utilization (edible parts and mode of utilization) variations of indigenous wild edible plants exist across the variety of regions and communities. These sorts of differences and similarities can be measured using the Jaccard Index (JI). The JI scores in the current study ranged from 35.3 to 1.1. (Table 3). The District Sargodha in Punjab [8], with a value of 35.3, recorded the highest value, followed by the Central Punjab, with a value of 24.3, and the District Jhelum, with a value of 13.5. All three sites are in Punjab. Due to similar geographic or climatic conditions, the greater JI score demonstrates the similarity in vegetation types between the two places. The minimum JI has been calculated in the study of Ishkoman and Yasin Valleys of Gilgit Baltistan, i.e., 1.1, the reported site is a mountainous region with a cold climate, whereas the current site is a plain area with a warm climate, and only one identical plant has been documented with the current site because of the stark differences in geography and climate between the two locations.

In terms of percentage similarity in edible part uses, the current study shows a greater similarity to data of the District Sargodha of Punjab (81.5%). This can be explained by the similar vegetation of both areas. District Sargodha’s similarity percentage also had greater resemblance with the data of district D.I. Khan of K.P.K. (Khyber Pakhtunkhwa Province), i.e., 72.7%. In terms of percentage similarity in the Mode of utilization, our study showed similarities to the data of District Sargodha and Central Punjab, i.e., 44.4 each, followed by the district D.I Khan of K.P.K (36.4.%). The lowest similarity was found to District Harnai of Balochistan (3.4), followed by Chitral of K.P.K (3.6), and Ishkoman and Yasin Valleys of K.P.K (3.7). The percentage of similarity for the mode of utilization identifies similarities or differences in how wild fruits and vegetables are used, including raw, cooked, pickled, sweet recipes, jams, beverages, sauces, tea, powder, etc., between the current results and the literature. The percentage similarity for both the part used and the mode of utilization was closer to other regions of Punjab than the other provinces of Pakistan because of cultural and traditional variations due to geographical and climatic differences among the communities of Punjab and other provinces where different communities use a plant with different parts and different modes. For example, in Punjab, *Ficus palmata* was used for its fruits only while in Gadoon valley and Lesser Himalays its leaves were eaten raw or in a cooked form.

The current study also sorted out 20 WFPs that had previously been rarely documented for human food from Pakistan, i.e., *Ehretia obtusifolia*, *E. accuminata*, *Opuntia stricta*, *Cleome brachycarpa*, *Mukia madraspatana*, *Croton bonplandianus*, *Euphorbia prostrata*, *Acacia nilotica*, *Pongamia pinnata*, *Grewia asiatica*, *Malvastrum coromandilianum*, *Morus serrata*, *Argemone mexicana*, *Bambusa vulgaris*, *Echinochloa colona*, *Trichosanthes dioica*, *Solanum surattense*, *Physalis minima*, *Withania somnifera*, *Fagonia cretica*, and *Peganum harmala*. However, of these, 19 WFPs have already been reported in the literature, but the current study highlighted 14 WFPs for their novel mode of utilization and 5 WFPs for their novel edible part usage.

## 4. Discussion

We investigated the role of WFPs in the nutrition and food system of five different ethnic populations living in the remote areas of semi-arid regions along the Pakistan–India border. Across the study region, vegetables were the most common WFP use category (with 35 different species). Other authors have conducted similar research on the dietary value of wild vegetables. For example, Abbas et al. [24] recorded 53 wild vegetables plants from Kurrum district. Similarly, Abbasi et al. [27] documented 45 wild vegetables from Lesser Himalayas. Due to higher rainfall and suitability for plant growth, the Kurram district and the area of the Lesser Himalayas have a richer diversity of wild vegetable species than our semi-arid region. While Ahmad et al. [23] reported 25 wild vegetable species from Northwest Pakistan, Aziz et al. [37] reported 21 vegetables used in the isolated Yasin and Ishkoman valleys of Gilgit Baltistan. The dry and harsh climatic conditions that characterize the semi-arid areas of the Indo–Pak border are strikingly similar to those in the Yasin and Ishkoman valleys and Northwest Pakistan. Therefore, a few wild vegetable species can be found in those areas. Another study documented 27 wild vegetables in the Hindu Kush Mountain Range along the Pakistan–Afghanistan border [3]. Tribal communities residing along the Pakistan–Afghanistan border use a variety of species not found in our study area, including *Caralluma tuberculata*, *Nasturtium officinale*, *Solanum villosum*, and *Descurainia sophia*. This variation in the utilization of wild vegetable species may be a result of the scarcity of food resources or racial disparities. We believe that the availability of wild vegetables, local climatic conditions, and ethnography influence how they are used in different regions. The majority of the wild vegetables observed are weeds, which may have been dominant in anthropogenic environments given their prevalence in many food ethnobotanies and a wide ecological range [38,39]. According to researchers, using wild food plants may be a more effective alternative for coping with food scarcity and achieving long-term nutritional purposes [40].

The second most significant plant component was fruit (25 species), which had a well-established place in food ethnobotany among various communities. It is generally accepted that people choose their fruit based on how strange or sweet it tastes [41]. Young people primarily gather fruits, which are then immediately consumed. Both men and women in the study area participate in the collection of WFPs, which for the majority of the plants occurs from March to June. Children typically eat the plants that are used as raw snacks. During the spring and early summer, WFPs are collected from canal banks, scrubland, farmlands, and backyard gardens; however, recent years have seen a significant decline in WFP consumption. Abbasi et al. [26], on the other hand, documented that tribal groups use 35 wild fruit species in the Lesser Himalayas, and Khan et al. [42] documented 47 wild fruit species from the Swat Valley. In comparison with the study region, the Lesser Himalayas and Swat Valley have more plant diversity and receive more precipitation during the monsoon season. Therefore, based on availability, climatic factors, nutritional value, and cultural knowledge, we propose that the diversity, distribution, and consumption of wild fruit species varies by geography.

We also found that several flavoring agents (condiments) were derived from plant species in traditional cocking, such as *Chenopodium album*, *Citrullus colocynthis*, *Trichosanthes dioica*, and *Mentha* species. Majeed et al. [8] recorded *Chenopodium album* as a vegetable, while in the current study the dried powder of the plant was used as a condiment for flavoring food. The people residing in remote areas collect the plant from the field and dry it at home and trade it to local markets to generate income. *Mentha* as a flavoring agent was also reported by Aziz et al. [13] from Gilgit Baltistan.

WFPs have been utilised as a long-standing alternate source of food and medicine in the region, according to survey participants, and are commonly harvested and used as raw snacks (particularly fruits, leaves, and seed portions), cooked as vegetables, fermented, and infused in hot water (i.e., herbal tea), as Sõukand and Kalle [43] also reported. Some WFPs (primarily *Chenopodium album*, *Citrullus colocynthis*, *Mentha*, *Trichosanthes dioica*, and *Solanum surattense*) are used as flavoring and coloring agents or spices to improve the nutraceutical potential of daily diets, demonstrating a strong link between cultural traditions and biodiversity. Many researchers from various regions reported similar types of unique blending [3,44]. The research area has a diverse and rich bio-cultural legacy as a result, and this paper might be helpful to future generations. In this study, similar to colleagues’ work, additional WFP characteristics were noted, including regional names, preferred plant parts, microhabitats, flowering months, and conventional culinary preparations [8,27,39]. Such WFP attributes allow for the better understanding of how closely culture and biodiversity are related. For instance, local names, microhabitats, and flowering months all aid in accurate identification, identifying potential occurrence sites, and determining the best time to collect WFPs. Similarly, the use of preferred WFP parts may differ from species to species, taste to taste, and culture to culture [7,8]. According to informants, the most preferred part was the fruit and participants reported that they frequently ate sweet fruits raw as snacks when they were available. Similarly, leaves were the second most popular plant part in the area due to their more pleasant flavor when cooked as a vegetable. The most frequently mentioned categories in terms of culinary preparations were raw snacks and cooked vegetables. Due to the strong connection between preferred plant parts and culinary preparations displaying rich traditional knowledge discovered in this study, local communities were also found to be knowledgeable about the best times to use local wild plant resources [45]. As a result of the study area’s remoteness and the majority of traditional knowledge related to WFPs being passed down from generation to generation, it was also shown that some local communities still preserve some distinctive bio-cultural heritage [12].

The adoption of a modern lifestyle by local and indigenous cultures is a significant contributor to the decline of traditional knowledge related to the collection and eating of WFPs [46]. Participants in the study observed that although until recently, local communities were still aware of the importance of home gardening, frequently communicated their traditional knowledge among themselves, and helped others in establishing home gardens to cultivate WFPs, this expertise is rapidly degrading as a result of socio-cultural adjustments to accommodate modern lifestyles and rising migration from rural areas to urban settlements. Our cross-cultural analysis showed heterogeneity in traditional knowledge linked to WFPs among the studied ethnic groups, and this might be due to social isolation, with people living in remote, isolated areas due to the border and river barriers and being endogamic. The differences in the food systems of the five ethnic groups support some degree of cross-cultural partition between Mewati, Rajpot, Dogar, Jutt, and Arain groups. The Arain ethnic group consumed eight unique species and its members still retain significant knowledge about WFPs found in pastures because they have a long history as pastoralists and still depend on WFP resources. Seven species were commonly used in three ethnic groups Jutt, Arain, and Dogar, the reason for this similarity being the long coexistence in the same geographical region. In contrast, the Mewati and Rajpot ethnic groups only migrated from India during the subcontinent partition. When comparing with cross-cultural studies in their respective regions, [12,13] also reported the same results. They also discussed the influence of geographical locations, seasonality, availability, cultural and religious similarities, and geographical resemblances.

In many nations, public health policies typically work under a concept of nutrition and food security that undervalues biodiversity and local populations’ traditional food ways [47]. Furthermore, industries and policies have largely failed to exploit these resources, resulting in wasted opportunities and the eventual adoption of more expensive or less sustainable, and often less nutritious, approaches. Many scientists and international conventions, including the Second Global Plan of Action for Plant Genetic Resources for Food and Agriculture, the International Treaty on Plant Genetic Resources for Food and Agriculture, and the Convention on Biological Diversity’s Global Strategy for Plant Conservation (CBD), have recognized the value and potential of WFPs for food and nutrition. We have identified food species that serve as food indicators, including *Amaranthus viridis*, *Physalis minima*, *Ziziphus nammularia*, *Chenopodium album*, *Cucumus melo*, and *Ficus palmata*, that are significant in regional diets, particularly during times of food scarcity brought on by disease or drought, acerbated by global environmental change. Due to their availability and accessibility, people on a low income often eat wild food species. However, there are a few aspects posing threats to the availability of these species, such as habitat fragmentation, heavy livestock grazing resulting in disturbance, and careless development. In order to sustain WFPs, more action is required at the local and national levels. This action must take advantage of the growing intercultural awareness in local communities. Through local knowledge and practices, this comprehensive approach to food biodiversity conservation enables the ongoing development and adaptation to socio-ecological change [26,48]. To assist nations and stakeholders in creating sustainable use plans, Borelli et al. [10] suggested a comprehensive strategy for utilizing and conserving WFPs. This strategy urges and directs the numerous stakeholders to take action in order to guarantee the long-term sustainability and upkeep of WFPs. The sustainability of use is an important consideration whenever wild plants are used and is especially important when they are promoted. The availability of these resources can be negatively impacted by overharvesting, eating whole plants, or using particular plant components, such as roots [2].

During the survey, we discovered that some wild food plants were being sold in the area. This ability to generate income for families and activate small-scale economies may also be critical for the long-term viability of these foraging practices. Furthermore, because they are affordable sources of nutrients, wild plant species can contribute significantly to the nutritional well-being of rural communities [49,50,51]. As a result, it is important to promote the potential nutritional benefits of wild vegetables in order to implement a more community-centered public health and nutritional strategy [52]. The current study can prevent the loss of local knowledge about these ingredients by providing baseline data for communities looking to implement food security and sovereignty. However, more fieldwork is required to fully comprehend the intricate interactions between livestock activities, traditional food heritage, and rural landscapes in other Pakistani “marginal” regions. The availability of wild food plants also decreased significantly in some contexts, according to our study participants, due to growing anthropogenic pressures, such as unsustainable farming conditions, the availability of cultivated vegetables, the penetration of industrial food, environmental change and degradation, cultural changes in the gendered division of labour, as well as the poor general governance of food policies and economies at both the regional and national levels [53,54]. Additionally, the younger generations are significantly and inexorably losing traditional knowledge. It is vital to assist people in rediscovering the knowledge and practices linked to local plant resources so they can use them for food, small-scale businesses, personal well-being, and as a response to the worrying population expansion and income decline seen in many parts of Pakistan and the rest of the globe. This goes especially for members of younger generations [55].

## 5. Conclusions

The local people of the district of Kasur, Punjab, Pakistan are from various ethnic groups and settled along the Indo–Pak border in the semi-arid region. This study documented 71 wild food plants, the majority of which were used as cooked vegetables (35 species) and raw snacks (25 species). *Amaranthus viridis*, *Physalis minima*, *Ziziphus nammularia*, *Chenopodium album*, *Cucumus melo*, and *Ficus palmata* are the food species that we have identified as food indicators and are important in regional diets, especially during times of food scarcity brought on by disease or drought. Due to their extensive history as pastoralists, the Arain ethnic group consumed eight unique species that still hold significant knowledge about WFPs found in pastures. The findings demonstrated that WFPs are still gathered and used in the area. Nineteen species had market value and supported regional economies in addition to providing valuable nutrition. The younger generations of remote ethnic groups had already less knowledge about WFP consumption, due to the influence of modernization, urbanization, and industrialization. More research is required to preserve this significant cultural gastronomic heritage. This study can be extremely important for maintaining and advancing the traditional knowledge of WFPs’ preparation and consumption. Future sustainable development initiatives aiming to develop bio-cultural safeguarding strategies in order to increase public awareness of the value of natural resources, including WFPs, may use the current research as a baseline. Future research should also perform thorough syntheses of food ethnobotanical data with historical and archaeobotanical data in order to comprehend human adaptation processes and the spatial and temporal dynamics of plant food ingredients among various human settlements in unexplored geographic areas.

## Figures and Tables

**Table 2:** Wild edible food plants based on nutritional values and economic uses by natives Pipli Pahar from the Indo–Pak border in Pakistan.

Family	Scientific Name	Indigenous Name	Growing Habit	Edible Part	Mode of Utilization and Recipes	Traded in Local Market	Seasonal Availability	Previous Reports in Pakistan
Aizoaceae	*Trianthema portulacastrum* L.	It Sit	Herb	Shoots	Shoots are cooked as vegetable. Fresh shoots are mixed with *spinacia oleracea* and are boiled in water and then fried in oil with onion, garlic, ginger.	No	March–April	1 2 3 ♦+4 5 6 7 8 9 10 11 12 ᴥ+13 14 15 16 17 18
Amaranthaceae	*Amaranthus viridis* L.	Chulai	Herb	Leaves	Leaves are cooked as vegetable	No	August–September	1 2 ♦+3 ᴥΨ 4 5 6 7 8 9 10 11 12 ᴥ+13 14 15 ᴥ+16 17 18
	*Digera muricata* (L.) Mart.	Tandla	Herb	Shoots	Shoots are cooked as vegetable.	No	August–September	1 2 ♦+3 ᴥΨ 4 5 6 7 8 9 10 11 12 ᴥ+13 14 15 ᴥ+16 17 18
Asteraceae	*Carthamus oxyacantha* M. Bieb.	Pohli/Chandni/Adhmaya/Barham Dandi	Herb	Whole Plant	Leaves with onion, garlic, ginger, and spices are cooked as vegetable. Whole plant is boiled in water to prepare herbal tea.	No	March–April	1 2 3 ᴥ+4 5 6 7 8 9 10 11 12 ᴥΨ13 14 15 16 17 18
	*Cichorium intybus* L.	Kasni	Herb	Shoots	Cooked as vegetable and boiled in water to prepare herbal drink.	Yes	March–April	1 2 3 ᴥ+4 5 6 7 8 9 ᴥ+10 11 12 ᴥ+13 ᴥ+14 15 ᴥΨ16 ᴥ+17 18 ᴥ+
	*Launaea procumbens* (Roxb.) Ramayya & Rajagopal	Bhattal	Herb	Whole Plant	Shoots are cooked as vegetable.	No	March–April	1 2 ᴥ+3 4 ᴥΨ5 6 7 8 ᴥ+9 10 11 12 13 14 15 ᴥΨ16 17 18
	*Sonchus oleraceus* (L.) L.	Dhudhak booti	Herb	Shoots	Fresh shoots are eaten raw. Cooked in boiled water and then fried in oil with onion, garlic, ginger, green chilly with spices.	No	February–April	1 2 ♦+3 4 5 6 7 8 9 10 ♦+11 12 13 14 15 ᴥΨ16 17 18
Boraginaceae	*Cordia dichotoma* G. Forst.	Lasoori	Tree	Fruits	Ripened fresh fruits are eaten raw. Unripe fruits are used to make Achaar (Pickle). Unripe fruits are ground with spices to prepare Chatney (Sauce).	Yes	March–April	♦Ψ1 ♦Ψ2 3 4 5 6 7 ♦Ψ8 9 10 11 12 13 14 15 16 17 18
	*Cordia myxa* L.	Lasoora	Tree	Fruits	Ripened fresh fruits are eaten raw. Unripe fruits are used to make Achaar (Pickle).	Yes	October–November	♦+1 ♦Ψ2 3 4 5 6 7 ♦Ψ8 9 10 11 12 13 14 15 16 17 18
	*Ehretia acuminata* R. Br.	Panna/Hanjair	Tree	Fruits	Ripened fresh fruits are eaten raw	No	June–July	♦Ψ1 ᴥ+2 3 4 5 6 7 8 9 10 11 12 13 14 15 16 17 18
	*Ehretia obtusifolia* Hochst. ex A. DC.	Gondhi	Shrub	Fruits	Ripened fresh fruits are eaten raw.	No	June–July	1 2 3 4 5 6 7 8 9 10 11 12 13 14 15 16 17 18 19
Brassicaceae	*Capsella bursa-pastoris* (L.) Medik	Saroo boti	Herb	Leaves, Seeds	Fresh leaves are cooked as vegetable. Oil from seeds used in cooking.	No	February–March	♦Ψ1 ᴥ+2 3 4 5 6 7 8 9 10 11 12 13 14 15 16 17 18
	*Sisymbrium irio* L.	Saro boti	Herb	Leaves	Fresh leaves are cooked as vegetable.	No	February–March	♦Ψ1 ᴥ+2 3 4 5 6 7 8 9 10 11 12 13 14 15 16 17 18
Cactaceae	*Opuntia stricta* (Haw.) Haw.	Chittar thor	Shrub	Fruit	Fresh fruit is eaten as raw	Yes	May–June	1 2 3 4 5 6 7 8 9 10 11 12 13 14 15 16 17 18
Cannabaceae	*Cannabis sativa* L.	Bhung	Herb	Leaves	Dried leaves are used as drink for cooling purpose.	No	March–April	1 2 ♦+3 4 5 6 7 8 9 10 11 12 13 14 15 16 17 18 19
Capparaceae	*Capparis decidua* (Forssk.) Edgew.	Dailay/Kariya	Shrub	Fruits	Unripe fruits are used to make Achaar (Pickle). Ripened fresh fruits are eaten raw. Ripened fruits are used to make Murabba (Jam).	No	August–September	♦Ψ1 ♦Ψ2 ♦Ψ3 4 5 6 7 ♦Ψ8 9 10 11 12 13 14 15 16 17 18
	*Cleome viscosa* L.	Bophali/Dophali	Herb	Shoots	Leave are cooked as vegetable.	No	March–April	1 2 3 4 5 6 7 8 9 10 11 12 13 14 15 16 17 18
Chenopodiaceae	*Chenopodium album* L.	Bathoo	Herb	Shoots	Fresh leaves are cooked as vegetable. Dried leaves powder used as condiment.	Yes	August–September	1 2 ♦+3 ᴥ+4 ᴥ+5 ♦+6 ᴥ+7 8 9 ᴥ+10 ᴥ+11 ᴥ+12 ᴥ+13 ♦+14 15 ᴥ+16 17 18 ᴥ+
	*Chenopodium murale* L.	Bathu/Karonda/Chulai	Herb	Shoots	Fresh leaves are cooked as vegetable.	No	March–April	1 2 ♦+3 4 5 6 7 8 9 10 ᴥ+11 12 13 ᴥ+14 15 16 17 18
Cucurbitaceae	*Citrullus colocynthis* (L.) Schrad.	Tumma/Kor Tumma	Herb	Fruits/Seeds	Unripe fruits are used to make Achaar (Pickle). Ripened fruits are used to make Jam. Dried seed are used for making powder used as condiment.	Yes	May–June	1 2 ♦Ψ3 4 5 6 7 8 9 10 ♦Ψ11 12 13 14 15 16 17 18
	*Cucumis melo* var. *agrestis* Naudin	Chibherr	Climber	Fruits/Seeds	Ripened fresh fruit are eaten raw. Fruits and seeds are used to prepared sauce by crushing with onions. Ripened fruits are also cooked as vegetable.	Yes	June–July	♦Ψ1 ♦Ψ2 ♦Ψ3 4 5 6 7 8 9 10 11 12 13 14 15 16 17 18
	*Momordica balsmina* L.	Jangli Karela	Climber	Fruits	Fresh fruit is cooked as vegetable	Yes	August–September	1 ♦Ψ2 3 4 5 6 7 8 9 10 11 12 13 14 15 16 17 18
	*Mukia madraspatana* (L.) M. Roem.	Chaina Chibherr	Climber	Fruits	Leaves are cooked as vegetable	No	May–June	1 2 3 4 5 6 7 8 9 10 11 12 13 14 15 16 17 18
	*Trichosanthes dioica* Roxb.	Chaminda	Climber	Fruit	Fresh fruit is cooked as vegetable. Dried fruit powder is used as condiment.	Yes	August–September	1 2 3 4 5 6 7 8 9 10 11 12 13 14 15 16 17 18
Euphorbiaceae	*Croton bonplandianus* L.	Langrol	Herb	Whole Plant	Plant is boiled in water and then sugar is added to make herbal tea.	No	March–April	1 2 3 4 5 6 7 8 9 10 11 12 13 14 15 16 17 18
	*Euphorbia prostrata* Aiton	Hazardani	Herb	Shoots	Fresh shoots are eaten raw.	No	March–April	1 2 3 4 5 6 7 8 9 10 11 12 13 14 15 16 17 18
Fabaceae	*Acacia farnesiana* (L.) Wild	Kiker	Shrub	Pods/Gum	Young pods are cocked as vegetable. Gum is used for making traditional foods.	No	March–April	1 2 3 4 5 6 7 8 9 10 11 12 13 14 15 16 17 18
	*Acacia nilotica* (L.) Delile	Desi Keekar	Tree	Pods/Flowers/Gum	Unripe pods are used to make Achaar (Pickle). Gum is mixed in water to make drink. Flowers are boiled in water to make herbal tea. Flowers are cooked as vegetable.	No	March–April	1 2 3 4 5 6 7 8 9 10 11 12 13 14 15 16 17 18
	*Bauhinia variegata* L.	Kachnar	Tree	Flowers/Pods	Buds are cooked as vegetable. Unripe buds and pods are used to make Achaar (Pickle).	Yes	October–November	♦+1 2 3 4 5 6 7 8 9 ♦Ψ10 11 12 13 14 ♦Ψ15 ♦Ψ16 17 18 ᴥΨ
	*Cassia fistula* L.	Amaltas/Ambartash	Tree	Fruits/Flowers/Seeds	Ripened fresh fruits are eaten raw. Leaves and flowers are cooked as vegetable.	Yes	October–November	♦Ψ1 2 3 4 5 6 7 8 9 10 11 12 13 14 15 16 17 18
	*Lathyrus aphaca* L.	Jangli matri		Leaves/Pods	Leaves and pods are cooked as vegetable.	No	March–April	1 2 3 4 5 6 7 8 9 10 11 12 13 14 15 16 17 18
	*Pongamia pinnata* L.	Karunjwa	Tree	Leaves	Leaves are used to make sauce.	No	March–April	1 2 3 4 5 6 7 8 9 10 11 12 13 14 15 16 17 18
	*Prosopis cineria* (L.) Druce	Jhand	Tree	Pods	Unripe pods are used to make Pickle.	No	March–April	♦Ψ1 2 3 4 5 6 7 8 9 10 11 12 13 14 15 16 17 18
	*Prosopis juliflora* (Sw.) DC.	Pahari Keekar/Jund/Arjun	Tree	Pods	Unripe pods are used to make Pickle. Pods are also cooked as vegetables.	No	March–April	1 2 ♦Ψ3 4 5 6 7 8 9 10 11 12 13 14 15 16 17 18
	*Rhynchosia minima* (L.) DC.	Jangli Matter	Climber	Leaves/Pods	Leaves and pods are cooked as vegetable.	No	August–September	1 2 3 4 5 6 7 8 9 10 11 12 13 14 15 16 17 18
	*Senna occidentalis* (L.) Link	Chasku	Herb	Seeds/Pods	Fresh pods and seeds are cooked as vegetable.	No	March–April	1 2 3 4 5 6 7 8 9 10 11 12 13 14 15 16 17 18
	*Vicia sativa* L	Jangli Rewari	Herb	Leaves/Pods	Fresh leaves and pods are cooked as vegetable.	No	March–April	1 2 3 4 5 6 7 8 9 10 11 12 13 14 15 16 17 18
Lamiaceae	*Mentha longifolia* (L.) L.	Podina	Herb	Leaves	Leaves are used as condiment and making herbal drink.	Yes	August–September	♦+1 ♦+2 3 4 5 6 ♦+7 8 ♦+9 10 11 12 13 ᴥΨ14 15 ᴥΨ16 ♦+17 ♦+18 ♦+
	*Mentha pulegium* L.	Nehri Podina	Herb	Leaves	Leaves are used as condiment and making herbal drink.	Yes	August–September	♦+1 ♦+2 3 4 5 6 7 8 9 10 11 12 13 14 15 16 17 18
Malvaceae	*Bombax ceiba* L.	Simbal	Tree	Roots	Young roots are used as vegetables	Yes	August-September	♦+1 ♦+2 3 4 5 6 7 8 9 10 11 12 13 14 15 16 17 18 19
	*Grewia tenax* (Forssk.) Fiori	Phalsa/Ganger	Shrub	Fruits	Ripened fresh fruits are eaten raw.	No	March–April	1 ♦+3 4 5 6 7 ♦+8 ♦+9 10 11 12 13 14 15 16 17 18
	*Malvastrum coromandilianum* (L.) Garcke	Cheri Choga	Herb	Seeds	Unripe seeds are eaten raw.	No	August–September	1 2 3 4 5 6 7 8 9 10 11 12 13 14 15 16 17 18
	*Malva parviflora* L.	Sonchal/Khubazi	Herb	Fruits/Leaves	Ripened fruits are eaten raw. Leaves are cooked as vegetable.	No	May–June	1 2 3 4 5 6 7 8 9 10 11 12 13 14 15 ᴥΨ16 17 18 ᴥΨ
Meliaceae	*Azadirachta indica* L.	Naim/Neem	Tree	Fruits/Leaves	Ripened fresh and dried fruits are eaten raw. Leaves are crushed and mixed in water to make herbal drink.	No	August–September	♦+1 ♦+2 3 4 5 6 7 8 9 10 11 12 13 14 15 16 17 18
Moraceae	*Ficus benghalensis* L.	Boher	Tree	Fruits	Ripened fresh fruits are eaten raw.	No	May–June	♦+1 2 3 4 5 6 7 8 9 10 11 12 13 14 15 16 17 18
	*Ficus palmata* Forssk.	Pakwara	Tree	Fruits	Ripened fresh fruits are eaten raw. Unripen fruit is cooked as vegetables	Yes	May–June	♦+1 ♦+2 3 4 5 6 ♦+7 8 ♦+9 10 11 12 13 ᴥΨ14 15 ᴥΨ16 ♦+17 ♦+18 ♦+
	*Ficus racemosa* L.	Gulhar	Tree	Fruits	Ripened fresh fruits are eaten raw.	No	May–June	♦+1 ♦+2 3 4 5 6 7 8 9 10 11 12 13 14 15 16 17 18
	*Ficus religiosa* L.	Peepal	Tree	Fruits	Ripened fresh fruits are eaten raw.	No	March–May	♦+1 ♦+2 3 4 5 6 7 8 9 10 11 12 13 14 15 16 17 18
	*Morus alba* L.	Toot	Tree	Fruits	Ripened fresh fruits are eaten raw.	Yes	March–April	♦+1 ♦+2 3 ♦+4 5 6 ♦+7 8 9 10 11 12 ♦+13 14 15 16 17 ♦+18 ♦+
	*Morus nigra* L.	Toot	Tree	Fruits	Ripened fresh fruits are eaten raw.	Yes	March–April	♦+1 ♦+2 3 ♦+4 5 6 ♦+7 8 9 10 11 12 ♦+13 14 15 16 17 ♦+18 ♦+
	*Morus serrata* Roxb.	Shahtoot	Tree	Fruits	Ripened fresh fruits are eaten raw.	Yes	March–April	1 2 3 4 5 6 7 8 9 10 11 12 13 14 15 16 17 18
Moringaceae	*Moringa oleifera* Lam.	Suhanjna	Tree	Fruits/Leaves/Flowers/Seeds/Roots	Ripened fresh fruits are cooked as vegetable. Unripe fruits and roots are pickled to make Pickle. Flowers and leaves are boiling in water to prepare herbal tea.	Yes	May–June	ᴥΨ 1 2 3 4 5 6 7 8 9 10 11 12 13 14 15 16 17 18
Myrtaceae	*Syzygium cumini* (L.) Skeels	Jaman	Tree	Fruits	Ripened fresh fruits are eaten raw.	Yes	May–June	♦+1 2 3 4 5 6 ♦Ψ7 8 9 10 11 12 13 14 15 16 17 18
Oxalidaceae	*Oxalis corniculata* L.	Khatti Mithi/Khatti Tee	Herb	Shoots	Fresh leaves are cooked as vegetable and for making sauce.	No	March–April	1 2 ᴥ+3 4 ᴥΨ5 6 7 8 ᴥΨ9 10 ♦Ψ11 12 ♦Ψ13 ♦Ψ14 ♦Ψ15 ♦Ψ16 ᴥΨ17 18 ᴥΨ
Papaveraceae	*Argemone mexicana* L.	Satyanasi/Dhatoora	Herb	Whole Plant	Plant is used for making herbal tea.	No	March–April	1 2 3 4 5 6 7 8 9 10 11 12 13 14 15 16 17 18
Poaceae	*Avena fatua* L.	Joun/Javi	Herb	Seeds	Seeds powder is used as flour for making bread.	No	May–June	♦Ψ1 2 3 4 5 6 7 8 9 10 11 12 13 14 15 16 17 18
	*Echinochloa colona* (L.) Link	Swanakh Grass	Herb	Seeds	Seeds are cooked or ground into flour to make bread.	No	May–June	1 2 3 4 5 6 7 8 9 10 11 12 13 14 15 16 17 18
Polygonaceae	*Polygonum plebeium* R. Br.	Hind Rani/Laal Jhaari.	Herb	Shoots	Leaves and stems are cooked as vegetables. Shoots are soaking in water to prepare cold drink.	No	August–September	1 2 ᴥΨ3 ♦Ψ4 5 6 7 8 9 10 ᴥΨ11 12 ♦Ψ13 14 15 16 17 18
	*Rumex dentatus* L.	Jangli Palak	Herb	Shoots	Fresh leaves are cooked as vegetable.	No	August–September	1 2 ♦+3 ᴥ+4 5 6 7 8 9 ᴥΨ10 ᴥ+11 ᴥ+12 ᴥ+13 ᴥ+14 15 ᴥΨ16 17 18 ᴥ+
Portulacaceae	*Portulaca oleracea* L.	Bathuwan Booti	Herb	Shoots	Shoots are boiled in water and then fried in oil with onion, garlic, ginger, green chilly with spices.	No	June–July	1 2 3 4 ♦+5 6 7 8 9 10 11 12 13 ♦+14 15 16 17 18 ♦+
Rhamnaceae	*Ziziphus mauritiana* Lam.	Beri	Tree	Fruits	Ripened fruits are eaten raw. Ripened fruits are dried for later use to make Joshanda (Herbal tea) with other dried herbs.	Yes	June–July	♦Ψ1 ♦+2 3 4 5 6 7 ♦Ψ8 9 10 11 12 13 14 15 16 17 18
	*Ziziphus nammularia* (Burm. f.) Wight & Arn.	Saho Berri	Tree	Fruits	Ripened fruits are eaten raw.	Yes	September–October	♦Ψ 1 2 ♦+3 ♦+4 5 6 ♦+7 ♦+8 ♦+9 10 11 12 ♦+13 14 ♦+15 16 ♦+17 ♦Ψ 18 ♦+
Salvadoraceae	*Salvadora oleoides* Decne	Waan	Tree	Fruits	Ripened fresh fruits are eaten raw.	No	June–July	♦+1 ♦+2 ♦Ψ 3 4 5 6 7 ♦+8 9 10 11 12 13 14 15 16 17 18
	*Salvadora persica* L.	Pilu	Tree	Fruits	Ripened fresh fruits are eaten raw.	No	June–July	♦+1 2 ♦Ψ 3 4 5 6 7 ♦+8 9 10 11 12 13 14 15 16 17 18
Solanaceae	*Solanum nigrum* L.	Peelakan	Herb	Whole Plant	Ripened fruits are eaten raw. Leaves are cooked as vegetable. Herbal tea is made by boiling the whole plant in water.	No	March–April	ᴥΨ1 ᴥΨ2 3 4 5 6 ᴥΨ7 8 9 10 11 12 13 ᴥΨ 14 15 ᴥΨ 16 ♦Ψ17 18 ᴥΨ
	*Solanum surattense* Burm. f.	Kandyari	Herb	Fruits/Leaves/Seeds	Dried seeds are used as condiment.	No	March–April	1 2 3 4 5 6 7 8 9 10 11 12 13 14 15 16 17 18
	*Physalis minima* L.	Bhamolay	Shrub	Fruits	Ripened fresh fruits are eaten raw.	No	April–July	1 2 3 4 5 6 7 8 9 10 11 12 13 14 15 16 17 18
	*Withania somnifera* (L.) Dunal	Asgandh Nagori/Paner dodhi/Rankan	Shrub	Fruits	Ripened fresh fruits are added in milk to make yogurt.	No	March–April	1 2 3 4 5 6 7 8 9 10 11 12 13 14 15 16 17 18
Zygophyllaceae	*Fagonia cretica* L.	Dhamman Dhamasa/Dhamaya	Herb	Whole Plant	Dried plant is grinded and taken in powder form. Fresh leaves are used to make cold drink. Herbal tea is made by boiling the plant in water and then adding sugar.	No	August–September	1 2 3 4 5 6 7 8 9 10 11 12 13 14 15 16 17 18
	*Peganum harmala* L.	Harmal	Herb	Whole Plant	Plant is cooked in water and milk with sugar and eaten as sweet dish.	No	March–April	1 2 3 4 5 6 7 8 9 10 11 12 13 14 15 16 17 18
	*Tribulus terrestris* L.	Bhakra	Herb	Seeds	Seed are cooked in oil for enhancing sexual power.	No	March–April	1 2 3 4 5 6 7 8 9 10 11 12 13 14 ᴥΨ15 16 17 ᴥΨ18

(♦) = Similar part use(s); (ᴥ) = Dissimilar part used; (+) = Similar mode of utilization; (Ψ) = Dissimilar or Multiple mode of utilization; ( )= Plants not reported in a previous study; 1: Shah et al. [17]; 2: Shah et al. [18]; 3: Majeed et al. [8]; 4: Tareen et al. [19]; 5: Aziz et al. [37] 6: Rashid et al. [20]; 7: Marwa et al. [21]; 8: Ahmad and Pieroni [22]; 9: Ahmad et al. [23]; 10: Abbas et al. [24]; 11: Aziz et al. [13]; 12: Abdullah et al. [3]; 13: Khan et al. [14]; 14: Shad et al. [28]; 15: Abbasi et al. [25]; 16: Abbasi et al. [26]; 17: Abbasi et al. [27]; 18 Iqbal et al. [7].

**Table 3:** Jaccard index comparing the present study with previous articles in the surrounding regions.

Author Citation	Study Area, Province	NP	NPSP	NPDP	NPSM	NPDM	TPCBA	PRAA	PRSA	PPSP	PPDP	PPSM	PPDM	JI
Shah et al. [17]	District Sargodha, Punjab	27	22	2	12	12	24	3	41	81.5	7.4	44.4	44.4	35.3
Shah et al. [18]	Central Punjab	27	17	1	12	6	18	9	47	63.0	3.7	44.4	22.2	24.3
Majeed et al. [8]	District Jhelum, Punjab	78	14	3	9	8	17	61	48	17.9	3.8	11.5	10.3	13.5
Tareen et al. [19]	District Harnai, Balochistan	59	1	3	2	2	4	55	61	1.7	5.1	3.4	3.4	3.3
Aziz et al. [37]	Ishkoman and Yasin Valleys, Gilgit Baltistan	27	1	0	1	0	1	26	64	3.7	0.0	3.7	0	1.1
Rashid et al. [20]	District Rajouri, Jammu Kashmir	57	6	2	6	2	8	49	57	10.5	3.5	10.5	3.5	7.0
Marwat et al. [21]	D.I. Khan, KPK	11	8	0	4	4	8	3	57	72.7	0.0	36.4	36.4	11.8
Ahmad and Pieroni [22]	Takhat Sulaiman Hills, KPK	51	3	2	4	1	5	46	60	5.9	3.9	7.8	2.0	4.5
Ahmad et al. [23]	Division (Malakand, Peshawar, Mardan, D.I. Khan, Bannu, Hazara and Kohat, KPK	25	1	4	3	2	5	20	60	4.0	16.0	12	8	5.9
Abbas et al. [24]	Kurram District, KPK	55	3	4	4	3	7	48	58	5.5	7.3	7.3	5.5	6.2
Aziz et al. [13]	Chitral, KPK	55	0	2	2	0	2	53	63	0.0	3.6	3.6	0	1.7
Abdullah et al. [3]	Hindu Kush Mountain Range KPK	63	5	6	8	3	11	52	54	7.9	9.5	12.7	4.8	9.4
Khan et al. [14]	Gadoon Valley	51	4	4	5	3	8	43	57	7.8	7.8	9.8	5.9	7.4
Shad et al. [28]	Different Mountain Packets, KPK	17	3	0	1	2	3	14	62	17.6	0.0	5.9	11.8	3.8
Abbasi et al. [25]	Lesser Himalayas, KPK and Punjab	35	6	0	4	2	6	29	59	17.1	0.0	11.4	5.7	6.4
Abbasi et al. [26]	Lesser Himalayas, KPK and Punjab	45	2	9	2	9	11	34	54	4.4	20.0	4.4	20	11.1
Abbasi et al. [27]	Fifteen different sites of Lesser Himalays, KPK and Kashmir	20	3	2	2	3	5	15	60	15.0	10.0	10	15	6.3
Iqbal et al. [7]	Western Himalaya, Azad Jammu & Kashmir	102	5	7	8	4	12	90	53	4.9	6.86	7.8	3.9	7.7

NP: Number of Total Plants, NPSP: Number of Plants with Similar Part Used, NPDP: Number of Plants with Dissimilar Part Used, NPSM: Number of Plants with Similar Mode of Utilization, NPDM: Number of Plants with Dissimilar or Multiple Mode of Utilization, TPCBA: Total Number of Plants Common in Both Area, PRAA: Plants Reported in Aligned Area, PRSA: Plants Reported in Study Area, PPSP: Percentage of Plants with Similar Part Used, PPDP: Percentage of Plants with Dissimilar Part Used, PPSM: Percentage of Plants with Similar Mode of Utilization, PPDM: Percentage of Plants with Dissimilar or Multiple Mode of Utilization, JI: Jaccard Index.

## Data Availability

Occurrence data are available on request to the first author.
